# The Role of Iron, Its Metabolism and Ferroptosis in Traumatic Brain Injury

**DOI:** 10.3389/fncel.2020.590789

**Published:** 2020-09-25

**Authors:** Sicheng Tang, Pan Gao, Hanmin Chen, Xiangyue Zhou, Yibo Ou, Yue He

**Affiliations:** ^1^Medical Clinic and Polyclinic IV, Ludwig-Maximilians-University Munich (LMU), Munich, Germany; ^2^Department of Translational Neurodegeneration, German Center for Neurodegenerative Diseases (DZNE), Munich, Germany; ^3^Department of Neurosurgery, Tongji Hospital, Tongji Medical College, Huazhong University of Science and Technology, Wuhan, China

**Keywords:** iron, ferroptosis, traumatic brain injury, iron metabolic disorder, iron chelator, reactive oxygen species

## Abstract

Traumatic brain injury (TBI) is a structural and physiological disruption of brain function caused by external forces. It is a major cause of death and disability for patients worldwide. TBI includes both primary and secondary impairments. Iron overload and ferroptosis highly involved in the pathophysiological process of secondary brain injury. Ferroptosis is a form of regulatory cell death, as increased iron accumulation in the brain leads to lipid peroxidation, reactive oxygen species (ROS) production, mitochondrial dysfunction and neuroinflammatory responses, resulting in cellular and neuronal damage. For this reason, eliminating factors like iron deposition and inhibiting lipid peroxidation may be a promising therapy. Iron chelators can be used to eliminate excess iron and to alleviate some of the clinical manifestations of TBI. In this review we will focus on the mechanisms of iron and ferroptosis involving the manifestations of TBI, broaden our understanding of the use of iron chelators for TBI. Through this review, we were able to better find novel clinical therapeutic directions for further TBI study.

## Introduction

Traumatic brain injury (TBI) is a serious contributor to the mortality and disability of adults worldwide. Just in China, population-based mortality of TBI is estimated to reach approximately 13 cases per 100,000 people (Jiang et al., 2019). Apart from direct injury due to trauma, patients usually have severe and extensive neuronal necrosis, brain tissue edema, breakdown of the blood-brain barrier (BBB), escalating oxidative stress, and overactive inflammation following TBI. A vicious cycle of these adverse factors culminates in death, disability, and vegetative state (Toklu and Tümer, 2015). Aging individuals in the context of TBI suffer a greater probability of cognitive impairment, dementia, and neurodegenerative disorders, which should also be responsible for the poor outcome of TBI patients. As such, this emphasizes the growing need for TBI therapeutic agents that are expected to mitigate all aspects of primary brain injury and eliminate the secondary pathological defects of TBI.

Iron homeostasis is gaining acceptance as an essential process in TBI pathology. Iron is a vital trace element for the maintenance of normal cellular physiology, plays a role in producing deoxyribonucleic acid (DNA) and adenosine triphosphate(ATP), joins in the tricarboxylic acid (TCA) cycle, functions as cofactor for various proteins in the electron transport chain, and is implicated in the synthesis of myelin phospholipids and multiple neurotransmitters in the central nervous system (CNS) (Ke and Qian, 2007; Ward et al., 2014).

Iron deficiency in the brain during lactation could also cause impaired neurotransmitter synthesis, resulting in delayed development of behavioral functions. Excess intracellular iron wreaks havoc on neurological functions, especially in the compromised brain under TBI. The destruction of the brain tissue, the collapse of the BBB and increased cerebral vascular permeability, and regional severe inflammatory reaction together lead to a large amount of iron rush from the blood into the brain parenchyma.

Excess iron, divalent ferrous ion Fe^2+^ precisely, can react with hydrogen peroxide (H_2_O_2_)or organic peroxide (ROOH) to yield soluble hydroxyl (HO) or lipid alkoxy (RO∙) radicals, respectively. This is the primary source of reactive oxygen species(ROS) produced by Fe^2+^ in the cell, known as the Fenton reaction (Dixon and Stockwell, 2014), which can eventually result in a new type of regulated cell death called ferroptosis characterized by iron-dependent lipid peroxidation. The neuronal membrane is rich in cholesterol and polyunsaturated fatty acids (PUFAs), which are highly susceptible to oxidation by ROS (Shichiri, 2014). In addition, the ability of neurons to scavenge ROS in an autonomous manner is limited, as reflected in decreased enzyme activities of superoxide dismutase(SOD) and glutathione peroxidase(GPX) in the brain compared to other types of tissue (Cand and Verdetti, 1989),and the unparalleled activities of SOD and GPX with the development of aerobic metabolism during postnatal maturation (Khan and Black, 2003). The evidence above deepens our impression that neurons are exceptionally vulnerable to iron overload.

Recent studies have reported that iron overload is strongly associated with the development of Alzheimer’s disease (AD) and Parkinson’s disease (PD). Iron overload is highly involved in the pathophysiological process of secondary brain injury reported in recent years (Imai et al., 2019). More importantly, iron deposition in the brain as an inevitable consequence of aging (Del et al., 2015) manifests a secure connection between impaired iron homeostasis and poor outcome of aging TBI patients. Here, we will discuss how the iron, as well as ferroptosis, which are involved in brain trauma, broaden our understanding of the role of iron metabolism played in TBI and explore a new means of effective treatment targeting iron and ferroptosis after TBI.

## Iron and Its Metabolism

Because of iron’s hydrophilic feature, the entry of iron from the circulating blood into the brain parenchyma requires effective conveyance through the BBB. Iron uptake across the BBB in adult animals is mediated by the transferrin receptor(TfR) on the proluminal surface of cerebral microvascular endothelial cells that line the BBB, and the transferrin(Tf)/TfR endocytosis approach is likely to be the primary route (Burdo et al., 2003; Ke and Qian, 2007). Lactoferrin (McKie et al., 2000) / lactoferrin receptor (LfR) and glycosylphosphatidylinositol (GPI) anchored melanotransferrin (MTf)/ secretory melanotransferrin may also serve in the iron passage through the BBB (Fillebeen et al., 1999; Moroo et al., 2003).

Ferric iron (Fe^3+^) is released in the endosome due to the acidic pH environment, and then is reduced into ferrous iron (Fe^2+^) in the endosome. Divalent metal transporter 1 (DMT1, also known as SLC11A2) mediates the release of Fe^2+^ from endosomes into labile iron pools of the cytoplasm (Ke and Qian, 2007). Ceruloplasmin, as a GPI-linked membrane protein, is expressed in the mammalian CNS, mainly in astrocytes that surround brain microvessels (Klomp et al., 1996). After the discovery of its ferroxidase activity, studies suggested that the role of ceruloplasmin in iron metabolism was to help export iron at the cellular level by binding with ferroportin (Attieh et al., 1999; Richardson, 1999).

The change of transferrin will affect the iron metabolism. Abnormalities in iron metabolism, including both iron deficiency and iron overload, will negatively affect the function of the whole body and lead to some diseases (Table 1). Iron deficiency decreases cytochrome oxidase activity in the brain, especially in the hippocampus and prefrontal regions (Carlson et al., 2007). It could lead to the disorders of brain development (Stugiewicz et al., 2016) such as motor development and cognitive memory (Wang et al., 2019). High levels of iron in the body can be deposited in the brain which is involved in some neurodegenerative diseases such as PD and AD (Harris et al., 1995; Andrews, 1999). The neurodegenerative disorder characterized by the accumulation of iron is also defined as neuroferritinopathy which is involved in TBI (Daglas and Adlard, 2018).

**TABLE 1:** Diseases related to the involvement of abnormal iron metabolism.

Iron metabolism	Causes	Diseases
Iron overload	1. Transfusion of the red blood cells 2. Intestinal absorption of iron 3. Genetic abnormalities of iron metabolism 4. Others	Transfusional siderosis Myelodysplastic syndromes Hereditary hemochromatosis Aceruloplasminemia Alzheimer’s disease Parkinson’s disease
Iron deficiency	1. Poor absorption (bowel resection, inflammatory) 2. Increase need for iron (pregnancy, blood donations, cancer of the stomach) 3. Decreased dietary iron intake 4. Genetic abnormalities of iron metabolism 5. Hemolysis	Iron deficiency anemia Iron-refractory iron deficiency anemia Anemia of chronic inflammation
Transferrin	1. Increase: deficiency of iron hypoxia 2. Decrease: dietary Immunologic stimulation Genetic abnormalities	Iron deficiency anemia Acute viral hepatitis Malignant tumor Rheumatoid arthritis Congential hypotransferrinemia Congenital atransferrinemia

## Ferroptosis and Its Molecular Mechanism

### Ferroptosis

Back in 2012, the Stockwell BR team defined novel regulatory cell death (RCD) as ferroptosis in a way that is completely different from other types of RCD (Table 2). The structural integrity of the nuclei in deceased cells is retained. Nuclear condensation or chromatin margination that usually arise in apoptosis, necrosis, and autophagy are not present in ferroptosis (Dolma et al., 2003). Under electron microscopy, ferroptosis contribute to cells death with exhibits shrunken mitochondria, increased mitochondrial membrane density, and loss of mitochondrial cristae (Yagoda et al., 2007; Dixon et al., 2012). Suppressing critical molecules in apoptosis, necrosis, and autophagy pathways failed to prevent this process, whereas selected antioxidants and iron chelators markedly alleviate this novel RCD (Dolma et al., 2003).

**TABLE 2:** Differences among ferroptosis, apoptosis and necroptosis.

Regulary cell death types	Morphology	Features	Marks
Ferroptosis	∙ Normal nuclear size ∙ Small mitochondria 1. High mitochondria membrane density 2. Reduction of mitochondria crista 3. Breakdown of outer mitochondria membrane ∙ Cell round up and float; Retained cell integrity	∙ Iron dependent ∙ Membrane lipid peroxidation	System X_c_^–^ GPX4 GSH VDAC2/3 TfR1 …
Apoptosis	∙ Nucleus 1. Reduction of nuclear volume 2. Nuclear fragmentation 3. Chromatin condensation ∙ Small cells; Cell membrane blebbing; Retraction of pseudopods	∙ Activation of caspases ∙ Oligonucleosomal DNA fragmentation	Bax Bak Bcl-2 Bcl-XL …
Necroptosis	∙ Nucleus: moderate chromatin condensation ∙ Swelling cell; Cell membrane rupture	∙ Slump in ATP levels ∙ Release of DAMPs	RIP1 RIP3 MLKL …

*GPX4: glutathione peroxidase 4; GSH: glutathione; VDAC2/3: Voltage-dependent anion channels 2/3; TfR1: transferrin receptor 1; Bcl-2: B-cell lymphoma 2; Bcl-XL: B-cell lymphoma-extra large; RIP: Receptor-interacting kinase; MLKL: mixed-lineage kinase domain-like protein.*

### Mechanism of Ferroptosis

**FIGURE 1:**
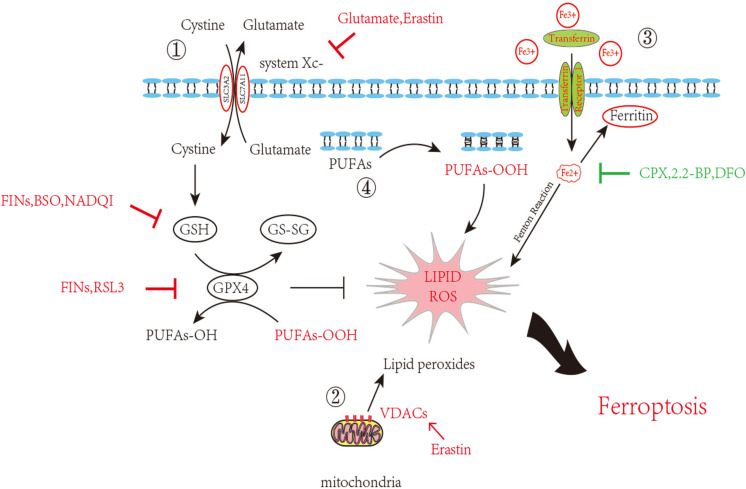
Inhibition of ferroptosis by inhibition of Xc^–^System or GPX4 activity. Scheme of several pathways implicated in ferroptosis in neurons. 1. The System X_c_^–^ pathway keeps the GSH maintenance, block of which can promote ferroptosis. GSH turning into GS-SG accompanied with PUFAs-OOH reduction and glutathione peroxidase 4 consumption. This process goes in a dynamic equilibrium, any perturbation on the component in this route such as GSH decrease, GPX4 depletion or increased external Glutamate will accelerate ferroptosis. 2. VDAC opening also contributes the ROS accumulation. 3. Iron overload brings into ROS through Fenton reaction. 4. Lipid peroxidation finally causes neuron death. Inducers and inhibitors of ferroptosis are marked in red and green, respectively. PUFAs: Polyunsaturated fatty acids; VDACs: Voltage-dependent anion channels/mitochondrial porins; GSH: Glutathione; GS-SG: Glutathione disulfide; GPX4: Glutathione peroxidase 4; FINs: Ferroptosis-inducing compounds; BSO: Buthionine sulfoximine; NADQI: N-acetyl-p-benzoquinone imine; RSL: RAS-selective lethal compounds; CPX: ciclopirox; 2,2-BP: 2,2-bipyridyl; DFO: deferoxamine.

As attention continues to be devoted to ferroptosis, the molecular mechanisms are being studied in depth. We list some molecular pathways that are now concretely proven to be engaged in ferroptosis (Figure 1).

#### System X_c_^–^-GSH Synthesis-GPX4

System X_c_^–^ is a Na^+^-dependent cysteine-glutamate antiporter located in the membrane. It exports intracellular glutamate to the extracellular space while importing cystine to the cytoplasm, whereupon cystine is subsequently converted to cysteine for glutathione (GSH) synthesis. Glutathione peroxidase 4 (GPX4) catalyzes GSH reaction with lipid–hydroperoxide to prevent ROS production, supporting cells against oxidative stress. System X_c_^–^, independent of ATP, is driven by high concentrations of intracellular glutamate; therefore, it is particularly sensitive to extracellular glutamate concentration. Millimolar extracellular glutamate can block system X_c_^–^ activity and initiate pathways of oxidative cell death previously known as oxytosis (Tan et al., 1999, 2001, Tobaben et al., 2011; Neitemeier et al., 2017). Elevated levels of extracellular glutamate in diverse brain trauma contexts inhibited system X_c_^–^ transportation, which in consequence triggered ferroptosis (Bridges et al., 2012). Recent studies suggest that directly inhibiting system X_c_^–^ can induce cell death by lowering uptake of cysteine, which leads to GSH depletion and ultimately impairs the cell’s ability to resist oxidative stress (Gout et al., 2001; Dixon et al., 2012).

GSH depletion following TBI is seen in several experiment cases and usually contributes to secondary injury (Bayir et al., 2002; Ansari et al., 2008). A number of other studies have also supported that GSH depletion is related to ferroptosis. N-acetyl-p-benzoquinone imine (NAPQI), an active metabolite of acetaminophen, First-class ferroptosis-inducing compounds (FINs) such as diverse pharmacological inhibitor-2 (DPI2), and Buthionine sulfoximine (BSO) all these compounds can deplete 90% of cellular GSH and can induce ferroptotic cell death (Yang et al., 2014; Lőrincz et al., 2015).

GPX4, one member of the GPX family, is found to be more involved in ferroptosis than the other counterparts. GPX4, together with GSH, reduces free hydrogen peroxide (H_2_O_2_) or organic peroxide (ROOH) into water or corresponding alcohols. GSH, at the same time, is turned into the oxidized counterpart glutathione disulfide (GSSG). GSH depletion induced by BSO deactivates GPX4, thereby elevating the ROS level in lipids, which is reflected by increased oxidation of NADPH and lysophosphatidylcholines (an indicator of lipid-producing ROS). Inhibition of GPX4 activity without any GSH deficiency also leads to ferroptotic cell death.

#### VDAC2/3

Voltage-dependent anion channels (VDACs), also referred to as membrane porins, are transmembrane channels that transport ions and metabolites in eukaryotes and are widely distributed on the external mitochondrial membrane. Knockdown of VDAC2/3, instead of VDAC1, suppressed erastin-induced death in RAS mutant cells. The affinity purification assay identified VDAC2/3 as a direct target of erastin, and the erastin-induced sensitivity strongly correlated with the amount of mitochondrial VDAC (Yagoda et al., 2007). Erastin acts by binding to VDAC2/3 on the external mitochondrial membrane, altering ion selectivity of the VDAC, so that the channel only permits cations to enter the mitochondria, retarding the oxidation of NADH, provoking mitochondrial dysfunction and oxidant release, and culminating in oxidation-dependent non-apoptotic cell death, now known as ferroptosis (Yagoda et al., 2007). Erastin additionally enhances the permeability of VDAC2 liposomes to NADH in a manner that requires the amino-terminal region of VDAC2 (Bauer et al., 2011).

#### Iron-Dependent Lipid Peroxidation

Overload of iron induces ferroptosis by the production of ROS via the Fenton reaction. This suggests that both increased iron uptake and decreased iron storage may account for the iron overload during ferroptosis. It is true that membrane-permeable iron chelators such as ciclopirox (CPX), 2,2-bipyridyl(2,2-BP) and impermeable iron chelators such as deferoxamine (DFO) provide adequate protection against ferroptotic cell death, while supplementation with exogenous iron sources or transferrin in combination with iron without treating with other divalent metals accelerates ferroptosis (Yagoda et al., 2007; Dixon et al., 2012). Iron-responsive element-binding protein 2(IREB2) is a major transcription factor in iron metabolism: applying RNAi repression of IREB2 considerably suppresses ferroptosis, providing direct evidence of iron-dependent cell death (Yagoda et al., 2007; Dixon et al., 2012). Suffice it to say that abnormalities in the metabolic system involved in iron acquisition and utilization are essential for the induction of ferroptosis.

The classic profile of ferroptosis is regulated cell death due to iron-dependent lipid peroxidation, which can be ameliorated by iron chelators and lipid antioxidants, and the culprit of lipid peroxidation is generally believed to be ROS. ROS can react with polyunsaturated fatty acids (PUFAs) of the membrane and induce lipid peroxidation. Several ROS-producing routes are described above (Figure 1); however, the detailed mechanism of ROS-induced iron death remains unclear. Some compounds that promote intracellular and mitochondrial ROS production fail to promote iron-dependent cell death, and hyperproduction of ROS is also known to be associated with other types of regulatory cell death (necrosis, apoptosis, etc.) (Dixon et al., 2012; Skouta et al., 2014, Su et al., 2019). An open question arises as to whether any type of lethal lipid peroxidation would be classified as ferroptosis, or whether only particular types of lethal lipid peroxidation would be referred to as ferroptosis. It is, therefore, of great demand to obtain an understanding of the specific lipids and their precursors participating in ferroptosis.

## The Role of Iron and Ferroptosis in TBI

TBI includes both primary and secondary impairments. Primary injury refers to fractures of the head caused by external forces. It produces cortical or subcortical contusions and lacerations, intracranial hemorrhage (subarachnoid hemorrhage or subdural hematoma) and disruption of the BBB. Diffuse axonal injury (DAI), the hallmark injury of TBI, is the main reason of some long-term complications of TBI which may be the reciprocal result of neuroinflammation and neurodegeneration (Ray et al., 2002). After trauma, axons in the white matter of the brain are more susceptible to damage. The main effects of DAI include primary mechanical rupture of the axonal cytoskeleton, which leads to disruption of axonal transport, resulting in axonal swelling and proteolysis (Johnson et al., 2013). Secondary injury refers to further molecular and chemical inflammatory reactions such as blood hemoglobin and iron release, dysfunction of brain metabolism and cerebral blood flow, further triggering neuroinflammatory processes, oxidative stress, glutamatergic excitotoxicity and mitochondrial dysfunction, etc., which cause further brain damage (Stocchetti et al., 2017).

**FIGURE 2:**
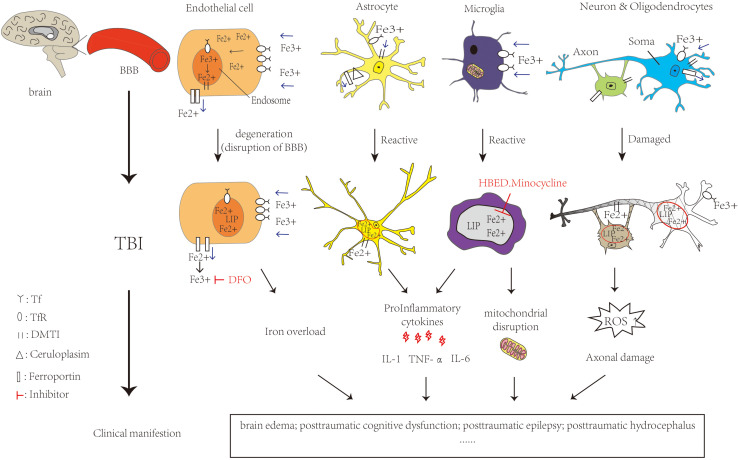
Iron metabolism in normal brain and cellular changes in the brain after TBI and the mechanism of iron chelators. Iron is taken up from the blood via endothelial cells in brain capillaries, Fe^3+^ binds to transferrin and transferrin receptors on endothelial cells entering the endosome through endocytosis. DMT1 can bind with Fe^2+^ to enter the LIP in the cytoplasm. Ferroportin is a transmembrane protein that can move Fe^2+^ out of the cell. In astrocytes, iron uptake occurs in the same way as in endothelial cells. Cp could efflux Fe^2+^ partnered with ferroportin. Oligodendrocytes also take up iron via the ferritin receptor Tim-2. Neuronal axons are surrounded by myelin sheaths, which are composed of oligodendrocytes in an iron-dependent manner. Microglia may take up iron via the transferrin receptor and release iron via ferritin. When TBI occurs, RBC in the capillaries rupture and hemoglobin is broken down by heme oxygenase, which can increase the NTBI. It is able to carry out Fenton reaction, making ROS increase. Meanwhile, the activation of astrocytes and microglia release pro-inflammatory cytokines and Fe^2+^ increased in the LIP. All of them cause oxidative damage or death to neurons and glia. The feature of ferroptosis is iron overload and ROS accumulation which lead to TBI physiopathology. Ferrostatin-1 is a specific ferroptosis inhibitor which alleviates ROS accumulation. The iron chelator DFO could bind to Fe^3+^. HBED crosses the BBB and promotes the oxidation of Fe^2+^ to Fe^3+^. Minocycline could also crosses the BBB and binds to Fe^2+^. All of them could reduce the extent of brain damage and limits the deterioration of brain function. The single arrow (pink) represents whether iron is transported into or out of the cell. DMT1: Divalent metal transporter 1; LIP: labile iron pools; Cp: ceruloplasmin; RBC: red blood cell; NTBI:Non-Transferrin Bound Iron fraction; ROS: reactive oxygen species; BBB: blood-brain barrier; DFO:deferoxamine; HBED: N,N′-di(2-hydroxybenzyl) ethylenediamine-N,N′-diacetic acid monohydrochloride.

Studies have reported that TBI patients who suffered from intracranial hemorrhage leading to the release of iron exhibited iron deposition in the brain. In TBI patients, microglia are activated to release toxic substances such as proinflammatory cytokines interleukin(IL-6) and tumor necrosis factor alpha (TNF-α), complement proteins and proteases causing brain damage (Dheen et al., 2007). At the same time, the BBB is disrupted, allowing further infiltration of neutrophils, macrophages, etc., into brain tissue (Chodobski et al., 2011). They are involved in cellular autophagy, secreting anti-inflammatory factors, enhancing the clearance of damaged cells and reducing the toxic effects caused by cellular degradation. Meanwhile, this could promote cellular repair and restore neuroplasticity (Krishnamurthy and Laskowitz, 2016). It will produce more toxic free radicals and ROS such as superoxide radicals and nitric oxide resulting in patients with cognitive dysfunction, brain edema and other hazards. With the generation of ROS, this results in reduced mitochondrial respiration, peroxidation of lipids, dysfunction of protein and enzyme oxidation, which could lead to neuronal damage (Fischer et al., 2016). Increased extracellular glutamate toxicity and overstimulation of glutamate N-methyl-D-aspartate (NMDA) and α-amino-3-hydroxy-5-methyl-4-isoxazolepropionic acid (Wilde et al., 2010) receptors can affect the intake of calcium and cause neuronal damage and cell death. TBI patients have increased extracellular glutamate at the cortex and hippocampus and decreased glutamate transporters. These changes lead to decreased GABAergic control and increased epileptic activity leading to cell death and affecting cognition (Palmer et al., 1993; Osteen et al., 2004) in the mouse model of TBI. Meanwhile it could lead to mitochondrial dysfunction and overproduction of free radicals, activate caspase signaling for apoptosis. We could find mitochondrial disruption, iron deposition and lipid ROS accumulation, all of them are characteristic of ferroptosis (Abdalkader et al., 2018). Figure 2 shows the iron metabolism in normal brain and cellular changes in the brain after TBI and the mechanism of iron chelators.

### Brain Edema After Trauma

Brain edema in TBI refers to a series of reactions caused by trauma, resulting in excess intracellular or extracellular water. It is a major complication of TBI patients. Meanwhile, the dense nature of the skull also leads to an increase in intracranial pressure (ICP), further contributing to the adverse consequences of TBI. Approximately 50% of TBI patients died from cerebral edema and its associated lesions (Marmarou, 2003; Simard et al., 2007). Timely alleviation of brain edema after TBI is helpful for patients’ recovery. Usually, the brain edema will peak within 2–3 days after TBI (Marmarou, 2003).

Brain edema in TBI is mainly divided into cytotoxic edema and vasogenic edema. In some TBI patients, the failure of the ATP-dependent Na^+^/K^+^ pump leads to an increase in intracellular ion content, osmotic pressure and the influx of water into the cells (Liang et al., 2007). Elevated extracellular K+ activates the Na^+^-K^+^-2Cl^–^ co-transporter (NKCC1), which facilitates Na^+^ entry into the cell and exacerbates brain edema (Zhang et al., 2017). Therefore, cytotoxic edema refers to an increase in intracellular water and no change in brain water content, which does not lead to an increase in ICP. Cytotoxic edema occurs in all cell types, especially in astrocytes, which predominantly causes swelling of the cells in the acute phase of TBI (Unterberg et al., 2004). The occurrence of cytotoxic edema may be related to aquaporin 4(AQP4), a bidirectional water channel protein that is widespread in astrocytes in the brain. Astrocytes play an important role in maintaining water and ion balance in the brain (Shields et al., 2011). Thus, AQP4 may be involved in the regulation of brain osmotic pressure gradients. Decreased AQP4 expression has been shown to reduce the formation of edema in TBI (Guo et al., 2006). IL-1β and TNF-α, as well as nuclear factor-kappa B (NF-κB), are all pro-inflammatory cytokines. TBI induces their upregulation, which is involved in the increased expression of AQP4 (Ito et al., 2006; Alexander et al., 2008, Rao et al., 2011). They are also involved in ferroptosis. Ferrostatin-1, the inhibitor of ferroptosis, further reduces the levels of IL-1β and TNF-α and BBB disruption, thereby reducing brain edema (Zhang et al., 2018). Meanwhile, TBI will cause the disruption of the balance between oxidative and antioxidant systems, which could also lead to brain edema (Hall et al., 2010). GPX4 is a major upstream regulator of ferroptosis, and its reduction increases ROS generation. The overexpression of GPX4 attenuates brain edema and BBB disruption in an animal model of TBI (Zhang et al., 2018). Arachidonic acid and other PUFAs will increase the amount of ROS, leading to ferroptosis. Increases in AA and PUFAs in the brain lead to increases in water and sodium and decreases in potassium and the ATP-dependent Na+/K+ pump, leading to cerebral edema (Chan et al., 1983).

Vasogenic edema is a disruption of the BBB that results in extravasation of fluid and intravascular proteins (such as albumin) into the brain parenchyma. The movement of water to the brain parenchyma creates a permeability gradient. The greatest feature of vasogenic edema is the traumatic opening of the BBB, where increased water content in the brain leads to swelling of brain tissue and increasing ICP (Barzó et al., 1997). Due to the peculiarities of the brain, the endothelial cells of the cerebral vessels are tightly interlinked by extracellular adhesion proteins. This forms the BBB, along with astrocytes and peripheral cells. After TBI, ischemia-reperfusion in the brain leads to mitochondrial dysfunction, glial cell activation, and excessive release of vascular permeability factors and cytokines (such as TNF-α and IL-6 and IL-1β) accompanied by oxidative stress, etc., leading to hyperpermeability and damage of the BBB (Jha et al., 2019). Vasogenic edema begins to appear a few hours after the onset of TBI (Barzó et al., 1997). Ferroptosis may be involved in vasogenic edema. Ferrostatin-1 and overexpression of GPX4 both reduce BBB destruction and alleviate vasogenic edema (Zhang et al., 2018). There are also some factors that cause vasogenic edema. Matrix metalloproteinases (MMPs) could degrade a variety of extracellular matrix proteins, including the tight junction proteins that make up the BBB. Substance P increases vascular permeability and activates astrocytes and microglia to produce pro-inflammatory factors, thereby increasing BBB destruction. Vascular endothelial growth factor A (VEGF-A) contributes to angiogenesis and increases microvascular permeability, and also has the ability to increase the permeability of brain endothelial cells. It has been found that MMPs, especially MMP-2, MMP-3, MMP-9, and VEGF-A, and substance P, are elevated after TBI, causing acute damage to the BBB, which leads to vasogenic edema (Hayashi et al., 2009; Jha et al., 2019).

With the help of magnetic resonance imaging (MRI), the increase in water diffusion distance indicated that vasogenic edema occurred in the first few hours after TBI, followed by cytotoxic edema persisting for 2 weeks that progressively worsened with increasing cellular dysfunction and death (Barzó et al., 1997). The destruction of BBB reaches its peak at 4–6 h after TBI, and differential permeability to molecules of different sizes is exhibited within 7 days. The destruction of the BBB further contributes to increased cytotoxic edema (Beaumont et al., 2000). Brain edema and ICP values peak within 2–3 days after TBI (Marmarou, 2003). Therefore, early intervention against angioedema and continued treatment of cytotoxic edema both contribute to patient prognosis. The treatment of brain edema mainly involves a combination of drugs and surgery, such as hyperosmolar treatment, hypothermia, sedation and decompressive craniectomy (Carney et al., 2017).

### Posttraumatic Cognitive Dysfunction

Cognitive dysfunction is mainly manifested as disturbed consciousness, memory disorders, attention deficit and impaired learning processing (Millis et al., 2001). Posttraumatic cognitive impairment can seriously affect quality of life. Patients with mild TBI may have transient consciousness impairment, mild confusion, inattention, and forgetfulness immediately after injury, with approximately 80%–85% of patients substantially recovering 3–6 months after injury (Dougan et al., 2014), and exhibiting better long-term prognosis. Only a small percentage of patients with mild TBI have cognitive impairment for a longer period of time, for several reasons (Vincent et al., 2014). Patients with moderate or severe TBI experience a loss of consciousness greater than 30 min or posttranmatic amnesia (PTA) (Gupta et al.) lasting more than 24 h. PTA refers to the time of confusion after TBI when a patient has a brief period of impaired consciousness and memory impairment. The duration of the PTA is very important to the patient’s prognosis. Patients with PTA often have more severe and persistent cognitive deficits after injury and may have more severe cognitive domain deficits involving language, reasoning, and visuospatial processing than patients with mild TBI. These patients are more likely to experience more prolonged impaired consciousness, such as coma or vegetative state, and are therefore at greater risk of developing chronic cognitive impairment after TBI (Nakase-Richardson et al., 2007). It has been reported that almost half of patients with moderate to severe TBI still have cognitive impairment even after 1 year, or even longer (Dougan et al., 2014). The age, gender and the presence of other chronic conditions such as diabetes, hypertension, etc., of TBI patients can affect the probability of cognitive dysfunction.

There are no definitive theories to indicate the cause of cognitive dysfunction, but some studies speculate that it is related to ferroptosis, DAI, neuronal deficits, and BBB disruption. A decrease in GSH was found in patients with TBI (Ansari et al., 2008), as well as iron deposition, which may be related to cognitive impairment (Maher, 2018). Administration of ferrostatin-1 to the ventricles of an animal model of TBI significantly reduces iron deposition and neuronal degeneration and improves cognition function, and thus ferroptosis may be involved in the process of cognitive impairment (Xie et al., 2019). Many studies have shown that head injury is a risk factor for AD (Fleminger et al., 2003). The greatest characteristic of AD patients is cognitive impairment, and neuronal deficits in AD, especially hippocampal neuronal damage with increased ferroptosis, which imply activation of the Nrf2/GPX4 signaling pathway, may also be the cause of cognitive impairment in TBI patients (Gómez-Isla et al., 1997; Yao et al., 2019). Meanwhile, animal models of AD have shown that BBB damage occurs early in AD patients (Zlokovic, 2011). Thus, damage to the BBB in TBI patients may also be a cause of cognitive dysfunction. DAI often occurs after TBI and is characterized by a loss of consciousness and extensive axonal damage to the cerebral hemispheres, cerebellum and brainstem. After TBI, the white matter axons of the brain are more susceptible to injury, and the shear force generated by head trauma on the white matter axons may cause them to break, which interrupts transport and causes swelling and other secondary physiological changes. Almost all patients with severe TBI showed DAI (Povlishock and Katz, 2005). It has been shown that in patients with TBI, intracerebral axonal deformities persist several years after injury and that DAI may induce long-term progressive neurodegenerative processes (Chen et al., 2009).

### Posttraumatic Epilepsy (PTE)

Posttraumatic Epilepsy is a common symptom after TBI and occurs in approximately 30% of patients with severe TBI (Bao et al., 2011). Approximately 80% of PTE patients have seizures within 2 years of TBI (Verellen and Cavazos, 2010). Severity of head injury, acute cerebral hematoma or subdural hematoma, diffuse hydrocephalus and penetrating brain injury are all risk factors for PTE (Curia et al., 2016). Posttraumatic seizure (PTS) is a seizure that occurs once after TBI due to head trauma, while PTE is a seizure that occurs multiple times at least one week after TBI. The features of PTE are recurrent epileptic symptoms with diverse clinical manifestations and a duration time ranging from weeks to months (Jennett, 1974; Raymont et al., 2010).

PTE is generally localization-related epilepsy caused by the temporal or frontal lobes (Gupta et al., 2014). It can further aggravate the symptoms of memory and cognitive impairment, sleep disorders, depression, etc., caused by TBI. It is thus necessary to diagnose and treat PTE in a timely manner, which can help patients to improve their quality of life (Castriotta et al., 2007; Muccigrosso et al., 2016). Computerized tomography (CT), MRI and electroencephalography (EEG) all contribute to the diagnosis of PTE. Antiepileptic drugs such as phenytoin, phenobarbital, levetiracetam and carbamazepine are all commonly used in clinical practice to prevent acute seizures after TBI. They are effective against early PTS, but the treatment for PTE is limited (Hung and Chen, 2012). If the intracranial seizure lesion causing the epilepsy can be identified, it can be treated with surgery. Different intracranial lesions require different surgical resection (Zimmermann et al., 2017).

The occurrence of PTE may be associated with damage to the BBB and intracranial hemorrhage caused by TBI. The infiltration of red blood cells and blood components causes hemolysis and hemoglobin breakdown, leading to the accumulation of free iron and iron-rich compounds such as heme. It has been shown that metals can stimulate the onset of epilepsy (Kendirli et al., 2014). Iron and ferrous ions initiate the inflammatory responses and enhance free radical production and mitochondrial fragmentation. The oxidative stress leads to lipid peroxidation, causing lipid damage which promotes epilepsy. This theory can be justified by the fact that injection of Fecl3 into mice could construct epilepsy models (Jyoti et al., 2009). In the TBI epilepsy model, the transcription factor Nrf2 involved in ferroptosis was significantly reduced, which promotes various expressions of antioxidant, anti-inflammation and neuroprotective proteins (Ghadiri et al., 2020). NMDA receptor-mediated glutamate excitotoxicity, ROS formation and subsequent membrane lipid peroxidation, and neuronal cell death all may exacerbate brain ischemia in TBI patients. Further depolarization leads to a lower seizure threshold, which induces epilepsy (Willmore and Ueda, 2009).

### Posttraumatic Hydrocephalus (PTH)

Posttraumatic Epilepsy is a special type of hydrocephalus that occurs after TBI and was first detected in children by Dandy in 1914 (Dandy and Blackfan, 1964). It is more common in the pediatric population (Fattahian et al., 2018). PTH occurs mainly within 3 months after TBI, and not all patients with TBI will have PTH, which is related to the patient’s age, degree of disability due to TBI, impairment of consciousness (Kammersgaard et al., 2013)and repeated operations (Choi et al., 2008). The cause of PTH may be related to intracranial hemorrhage and adhesion of inflammatory mediators that affect the outflow and resorption of cerebrospinal fluid (CSF) after TBI (Miki et al., 2006; Shah and Komotar, 2013). The occurrence of PTH may also be related to an excess of iron in the brain. One study found that injection of red blood cells into the cisterna magna of dogs led to an increase in iron and the formation of hydrocephalus (Iwanowski and Olszewski, 1960). A study showed that SAH patients had significantly elevated levels of iron and ferritin in their CSF.

Patients with SAH after TBI are more likely to develop PTH and are at three times greater risk than patients with other types of TBI (Chen et al., 2019). The increase in ferritin may be associated with the stronger inflammatory response in the subarachnoid space, leading to an increase in inflammatory cells and the promotion of ferritin synthesis by inflammatory cytokines. Meanwhile, it has been shown in animal models that injection of hemoglobin leads to increased activity of heme oxygenases (HOs) accelerating heme metabolism (Turner et al., 1998), increasing erythrocyte lysis and leading to an excess of iron in the brain. Iron can cause ventricular expansion. The iron chelator deferoxamine (DFO), which could upregulate HO-1, can thus alleviate ventricular expansion caused by TBI leading to acute hydrocephalus (Zhao et al., 2014). DFO could bind and remove iron from ferritin in a concentration-dependent manner, and remove approximately 10–15% iron from saturated transferrin, but none from hemoglobin (Zhao et al., 2014). Increased lipid peroxidation leads to vascular changes that can also cause hydrocephalus (Caner et al., 1993).

Posttraumatic Epilepsy can be manifested as normal pressure hydrocephalus (NPH). Its typical symptoms are cognitive impairment, urinary impairment and gait changes. It can also be manifested as hypertrophic hydrocephalus with increased ICP, manifested as headache, nausea, vomiting, optic papilledema, focal neurological dysfunction, etc. (Williams and Malm, 2016). A large proportion of patients are difficult to detect because the symptoms of TBI are more consistent with those of PTH, with a wide variation in diagnosis, with incidence rates ranging from 0.7% to 51.4% (De Bonis et al., 2010). The imaging of PTH can sometimes present as acute obstructive hydrocephalus with enlargement of the ventricular system, especially in the anterior horn of the lateral ventricles; a clear band of interstitial edema around the lateral ventricles, especially in the frontal horn; enlargement of the ventricles more than the cistern; no atrophy in the gyri and no widening of the sulcus (Kishore et al., 1978). The frontal horn index is the ratio between the maximum width of the frontal horn and the maximum width of the brain in the same axial CT section. It has been reported that a ratio greater than 0.3 indicates enlarged ventricles (Kosteljanetz and Ingstrup, 1985).

## Iron Quantification Measurement in TBI

Thanks to the development of MRI techniques, more new methods are being introduced every year. Some of these have existed for decades, but because their function has not been fully discovered, they generally require special manipulation and evaluation. Therefore, they are still considered advanced techniques.

With iron accumulation playing a role in TBI pathology, magnetic field-related MR imaging is sensitive to the presence of non-heme iron (Wu et al., 2010; Raz et al., 2011), and some MR imaging techniques can better detect and assess the correlation between iron deposition and patients with TBI.

Iron accumulation in specific areas of the brain in TBI patients can be recorded by MRI approaches such as magnetic field correlation (MFC) and susceptibility weighted imaging (Dougan et al., Raz et al., 2011). MFC is the imaging metric of MRI with the quantitative value of magnetic field in homogeneities (MFIs). The values of MFIs are varied according to the different magnetic sensitivities of different tissues in MRI and different structures of macro and micro tissues (Jensen et al., 2006). MFC values are increased in iron-rich cells. Therefore, MFC can be used as a biomarker of iron in MRI. It has been shown that MFC values are significantly elevated in the thalamus and globular pallidum and deep gray matter of TBI patients, suggesting that iron is involved in both white matter and subcortical gray matter damage in TBI formation (Raz et al., 2011). Susceptibility Weighted Imaging (SWI) is particularly sensitive to changes of iron and blood oxygen saturation in the brain, thus showing the magnetic susceptibility changes of different tissues (Liu et al., 2016). It has been shown that SWI can detect iron levels in the brain and determine the severity of the diseases in PD patients (Wu et al., 2014). SWI performed on TBI patients reveals elevated angle radian values in intracerebral gray matter and subcortical gray matter (Lu et al., 2015). Diffusion tensor imaging (DTI) metrics can reflect subtle changes in the white matter of the brain due to demyelination or disruption of tissue microstructure in the TBI patients, resulting in decreased fractional anisotropy and increased mean diffusivity (Wilde et al., 2010; Wallace et al., 2018). DTI is more sensitive than MRI to minor changes in mild TBI (Shenton et al., 2012).

## Drug Treatment Targeting Iron and Ferroptosis After TBI

Currently, TBI treatment is mainly divided into medical interventions and surgical treatments. Medical interventions include monitoring intracranial pressure and elevating the patient’s head during hyperventilation, which can lower the patient’s ICP (Greenberg and Arredondo, 2001), maintain hypothermia and reduce oxidative damage (Bayir et al., 2009). Intravenous mannitol is administered to alter blood rheology by hypertonic therapy (Golden et al., 2002). Hyperbaric oxygen therapy can improve cerebral blood flow in patients with both mild or severe TBI, and it could lead to a better quality of life (Lv et al., 2011). Performing the operations of removing hematoma and decompressive craniectomy in a timely manner can effectively improve the patient’s survival rate if the patient has epidural, subdural or intracranial hematoma. Iron overload leads to an increase in ROS, oxidative damage, and destruction of neural tissue-associated enzymes and cells, thus affecting the recovery of TBI patients. Iron chelators may thus represent a new direction for the treatment of TBI.

The therapeutic principle of iron chelators is to combine free iron and remove excess iron from the blood, while acting as an oxidant and promoting the generation of free radicals (Khalaf et al., 2018). Although there are many types of iron chelators, all of them contain oxygen, nitrogen or sulfur donor atoms that bind to iron to form dative covalent bonds. Ligand iron chelators containing “soft” donor atoms such as sulfur or nitrogen bind more readily to Fe2+ (Liu and Hider, 2002). Ligand chelators containing “hard” ligand atoms, such as oxygen, are more likely to bind to Fe3+ (Kalinowski and Richardson, 2005). Different iron chelators have different affinities with iron; the highest affinity iron chelator is hexadentate, such as DFO, which binds iron in a 1:1 ratio (Hider and Zhou, 2005). Iron chelators were initially used as adjuncts to the treatment of β-thalassemia, sickle cell disease and myelodysplastic disorders (Hatcher et al., 2009; Bayhan et al., 2018). There is also a small amount of clinical research on the treatment of AD (Crapper McLachlan et al., 1991) and PD (Devos et al., 2014). At present, there is no research on the treatment of TBI patients with iron chelators, but in many animal TBI models, there are already related applications.

There are currently three main types of medical iron chelators: siderophores, synthetic chelators, and natural-derived chelators (Hatcher et al., 2009). Siderophores are low molecular weight compounds extracted from microorganisms, and the most commonly used medicinal siderophore is DFO (Hatcher et al., 2009). DFO acts as a non-transferrin chelator to reduce hemoglobin by ferryl reduction and to produce superoxide radicals by binding to Fe3+. It also acts as a reducing agent to prevent lipid peroxidation. DFO also reduces hydrocephalus formation and HO-1 expression in a fluid percussion injury model (Zhao et al., 2014). In the weight loss animal models, DFO reduces iron and ferritin levels, improves spatial memory and reduces the possibility of brain atrophy (Zhang et al., 2013). In an animal model of intracranial hemorrhage, DFO has beneficial effects on iron-induced cerebral edema (Xie et al., 2014). N, N’-di(2-hydroxybenzyl) ethylenediamine-N,N’-diacetic acid monohydrochloride (HBED) is also an iron chelator which binds to ferrous iron through the BBB, converting it to ferric iron and mitigating iron’s harm. Compared with desferrioxamine, HBED is a synthetic product which has a higher affinity toward iron and fewer side effects (Bergeron et al., 1998). In animal models, HBED reduces the extent of brain damage and limits the deterioration of brain function after a TBI. It could improve motor function and neurological dysfunction, reducing the amount of cortical damage and oxidative stress markers (Khalaf et al., 2018). This was accompanied by a significant reduction in brain injury volume and hippocampal swelling. HBED could also decrease the expression of the AQP channel, which is involved in cell edema, and could reduce brain edema after TBI (Hatcher et al., 2009). Minocycline is a highly lipophilic compound and an iron chelator that crosses the BBB easily. It has a higher activity in reducing iron-induced injury compared to DFO (Garton et al., 2016). In TBI animal models, minocycline was shown to reduce iron overload, neuronal death and lower serum iron levels. It provides neuroprotection by inhibiting both the activation of microglia and inflammatory response (Murata et al., 2008) and attenuates iron-induced cerebral edema and BBB damage after intracerebral hemorrhage (Zhao et al., 2011). It has also been shown in clinical trials that minocycline has an effect on outcome in patients with hemorrhage and TBI.

Ferrostatin−1 (Fer-1) is a specific ferroptosis inhibitor that reduces cell death in some models such as Huntington’s Disease, acute brain injury, and so on (Jin et al., 2015). It has been shown that Fer-1 not only reduces iron aggregation and reduces neuronal death in TBI animal models but also improves TBI-induced cognitive and motor function deficits (Abdalkader et al., 2018). In addition, there are some traditional Chinese medicines that may play a role in the treatment of TBI. Baicalein, a flavonoid compound, can have a neuroprotective effect in many neurological diseases (Gu et al., 2016). In an animal model of FeCl_3_-induced PTE, it ameliorates behavioral seizures and exerts neuroprotective effects by inhibiting 12/15-LOX and preventing lipid peroxidation, thereby inhibiting ferroptosis to some extent (Li et al., 2019).

## Conclusion and Perspectives

It is becoming increasingly clear that iron and ferroptosis play a detrimental role in the pathogenesis of TBI. As a novel form of cell death, ferroptosis occurs in cells when iron accumulation and lipid peroxidation are activated. However, our understanding of these processes in TBI remains at an early stage. It is necessary to explore the relationship of iron with the primary and secondary consequences of TBI. More studies on the specific regulatory mechanisms of ferroptosis on clinical manifestations in TBI and the role of iron in ROS generation and neurotoxicity need to be conducted. Currently, due to the toxicity to both kidney and heart, iron chelators are only used experimentally. The clinical application of iron chelation therapies on TBI patients, and the long-term prognosis, are also worthy of further study.

## Author Contributions

ST and YH conceived the main outline. ST wrote the manuscript. PG and HC made the figures. XZ and YO took charge for manuscript revision in English. YH and YO participated in the correction and finally review of this manuscript. All authors contributed to the article and approved the submitted version.

## Conflict of Interest

The authors declare that the research was conducted in the absence of any commercial or financial relationships that could be construed as a potential conflict of interest.
